# Case Report: Successful transcatheter repair of left ventricle-right atrium connection following ventricular septal defect surgery

**DOI:** 10.3389/fcvm.2025.1667869

**Published:** 2026-01-16

**Authors:** Damba Dwisepto Aulia Sakti, Yovi Kurniawati, Rina Ariani, Leroy Leon Leopold Lasanudin, Radityo Prakoso

**Affiliations:** 1 Department of Cardiology and Vascular Medicine, Faculty of Medicine Universitas Indonesia, National Cardiovascular Center Harapan Kita, Jakarta, Indonesia; 2 Faculty of Medicine, Universitas Indonesia, Jakarta, Indonesia

**Keywords:** Konar-MF occluder, left ventricle-right atrium connection, pediatric, retrograde approach, transcatheter repair, tricuspid leaflet defect, ventricular septal defect (VSD)

## Abstract

**Background:**

Left ventricle-right atrium (LV-RA) connection may develop secondary to ventricular septal defect (VSD) repair, particularly when the membranous septum or tricuspid valve is affected, leading to a leaflet defect. While surgery remains the standard treatment, reports of transcatheter valve closure are limited. This highlights the importance of exploring the safety and feasibility of transcatheter approaches for LV-RA connection, especially in high-risk patients.

**Case presentation:**

This is the first reported case of a retrograde Konar-MF closure of an LV-RA connection on a 6-year-old male patient with a history of VSD surgery. Pre-procedural transesophageal echocardiography (TEE) showed a 4 mm defect in the septal leaflet of the tricuspid valve. A residual membranous septal aneurysm (MSA) extending from the subaortic region to the septal annulus of the tricuspid valve without residual shunt, and a small atrial septal defect (ASD) were also identified. The cardiac team performed closure using Konar-MF VSD Occluder No. 6/8 mm via a retrograde right femoral arterial approach, advancing the catheter through the left ventricle and across the defect. A well-positioned device with minimal residual central shunt and without peripheral leakage were confirmed from the post-procedural TEE. Clinical evaluation immediately after procedure and at 3-month follow-up demonstrated good results.

**Conclusion:**

Percutaneous transcatheter device closure of an LV-RA connection in pediatrics is a feasible alternative to surgery using a retrograde technique.

## 1 Introduction

Left ventricle-right atrium (LV-RA) connection is a rare cardiac anomaly that allows blood to flow from the left ventricle to the right atrium. This condition may be congenital or acquired. Congenital forms, classically referred to as a Gerbode defect, manifest as a malformation in the membranous atrioventricular septum (1). In contrast, acquired cases often develop secondary to an iatrogenic injury. Postoperative LV-RA shunt is a recognized complication following a ventricular septal defect (VSD) repair, especially when it involves manipulation of the membranous septum or tricuspid valve apparatus leading to a leaflet defect.

Defects of valve leaflets are typically seen in high pressure chambers of the heart, i.e., the mitral or aortic valve. Their susceptibility towards infective endocarditis presents as the most frequent cause (2). In cases where the tricuspid valve is affected, defects such as leaflet perforations are often associated with implanted pacemaker or defibrillator leads (3, 4). This results in tricuspid regurgitation, and ultimately progress to right-sided heart failure, if left unrepaired.

The current mainstay treatment option for valve leaflet defects is surgery. However, successful percutaneous transcatheter closures have been documented in multiple cases of the mitral valve, and should be considered as a viable alternative, especially in high-risk populations (5–9). The scarcity of reports on LV-RA connection due to tricuspid valve defects highlights the critical need to explore its safety and feasibility. In this report, we present a pediatric case of percutaneous transcatheter device closure of an LV-RA connection following a VSD surgical closure.

## 2 Case description

### 2.1 Presentation

**Figure 1:**
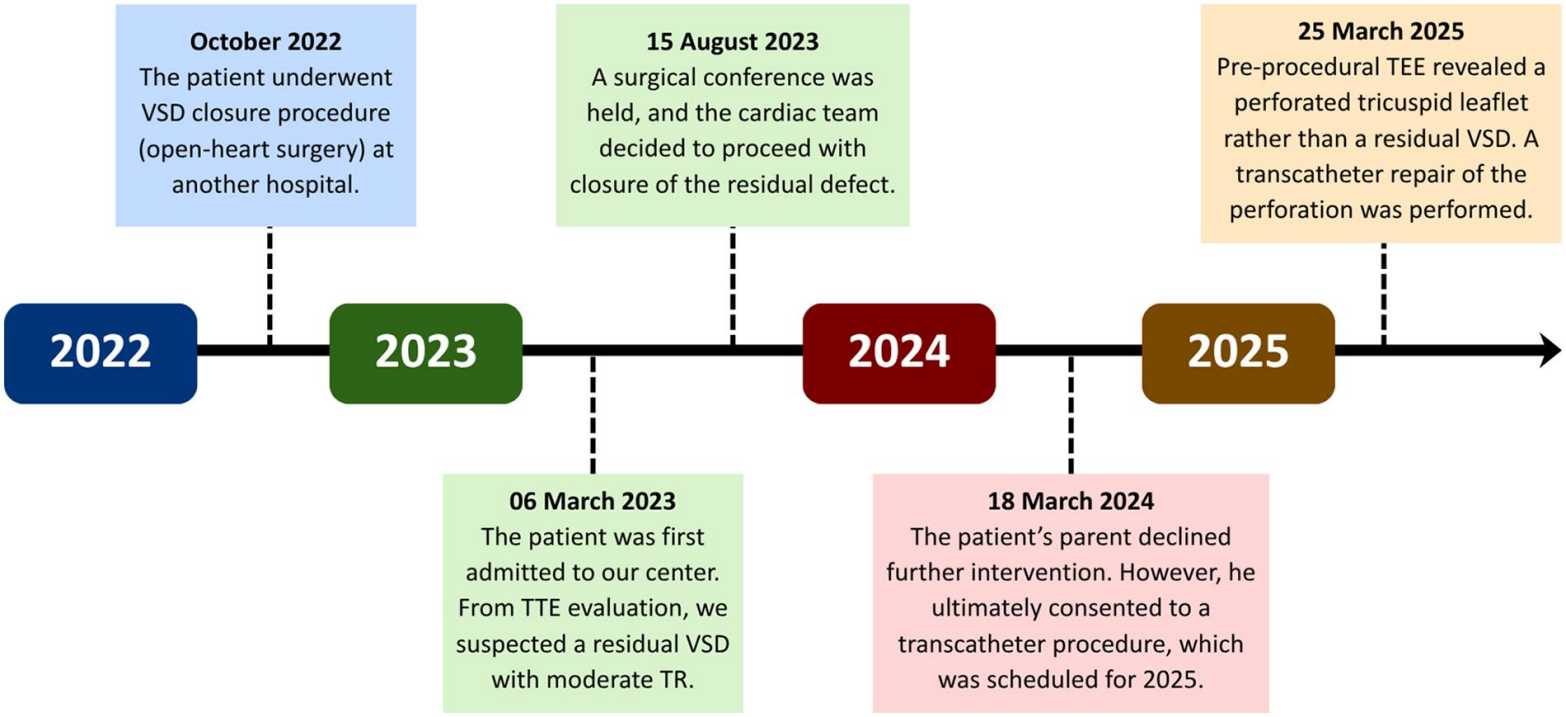
Timeline of patient care. TEE, transesophageal echocardiography; TTE, transthoracic echocardiography; TR, tricuspid regurgitation; VSD, ventricular septal defect.

A 6-year-old male patient with a history of VSD closure in another hospital at the age of 3 years and 10 months (October 2022) was referred to our center in March 2023 for further evaluation of suspected residual VSD shunt and the possibility for a transcatheter closure. The patient was asymptomatic on admission, and was given a routine therapy of sildenafil 3 × 15 mg and ramipril 1 × 2.5 mg from the previous hospital. There was no prior history of fever or other signs alluding to infective endocarditis. At our center, transthoracic echocardiography (TTE) demonstrated a systolic jet flow directed towards the right atrium. This was initially suspected as tricuspid regurgitation; however, the possibility of a residual VSD could not be ruled out. A surgical conference was then carried out in August 2023 with the decision to evaluate the flow ratio, followed by a device closure. Upon further assessment, a tricuspid leaflet defect was discovered from pre-procedural transesophageal echocardiography (TEE), leading to a transcatheter repair of the valve. An overview of the patient's timeline of care is summarized in Figure 1.

### 2.2 Physical examination

On the day of the procedure, the patient weighed 17 kg and was 108 cm tall (Supplementary Figure 1). Vital signs were normal with blood pressure of 113/64 mmHg, pulse rate of 91 beats per minute, and 98% blood oxygen saturation on room air. A pansystolic murmur grade II/VI was heard at the lower left sternal border.

### 2.3 Diagnostic assessment

Electrocardiogram readings revealed sinus rhythm with right axis deviation, right ventricular hypertrophy, and right bundle branch block (Supplementary Figure 2). On chest x-ray, cardiomegaly (cardiothoracic ratio of 62%) with an upward-pointing apex, dilated aortic segment, prominent pulmonary segment, and pulmonary plethora were observed (Supplementary Figure 3).

TTE evaluation in March 2023 revealed a normally positioned heart with atrioventricular and ventriculoarterial concordance. All pulmonary veins drained into the left atrium. There was no evidence of atrial septal defect (ASD) or patent ductus arteriosus. A small echo gap suspected as residual VSD was detected near the tricuspid valve, measuring at 3 × 5.5 mm with unclear flow of jet. Left ventricular systolic function was preserved with an estimated ejection fraction of 64%. Right ventricular contractility was also adequate, with a tricuspid annular plane systolic excursion of 13 mm. Heart valves were normal except for a jet directed towards the right atrium, which was interpreted as tricuspid regurgitation with a gradient of 80 mmHg. Further TEE examination revealed that the jet originated from the left ventricle to the right atrium with no VSD (Supplementary Videos 1, 2, 3).

### 2.4 Procedure

**Figure 2:**
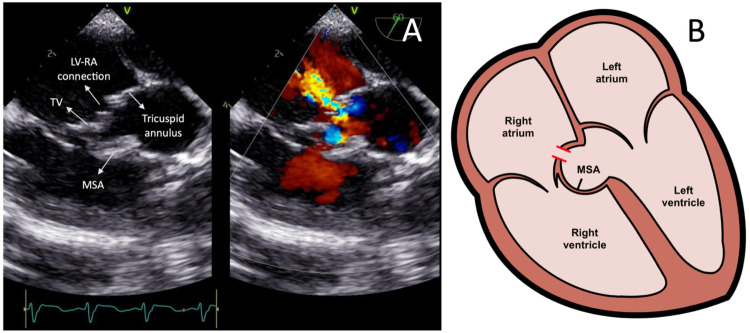
**(A)** Transesophageal echocardiography images showing the left ventricle-right atrium connection prior to the device closure. **(B)** Heart illustration representing the left ventricle-right atrium connection from the tricuspid valve leaflet defect and associated membranous septal aneurysm. LV-RA, left ventricle-right atrium; MSA, membranous septal aneurysm; TV, tricuspid valve.

Transcatheter closure of the LV-RA connection was carried out in 2025 following the patient's parent's consent. Pre-procedural TEE showed a 4 mm defect in the septal leaflet of the tricuspid valve, initially misinterpreted as a residual VSD (Figure 2A), creating moderate shunt from the left ventricle to the right atrium. A residual membranous septal aneurysm (MSA) extending from the aortic region to the septal annulus of the tricuspid valve was also observed, without any evidence of a residual VSD shunt to the right ventricle (Figure 2B). Additionally, a small left-to-right ASD was identified. A TEE-guided tricuspid defect device closure was decided using Konar-MF VSD Occluder No. 8/6 mm.

**Figure 3:**
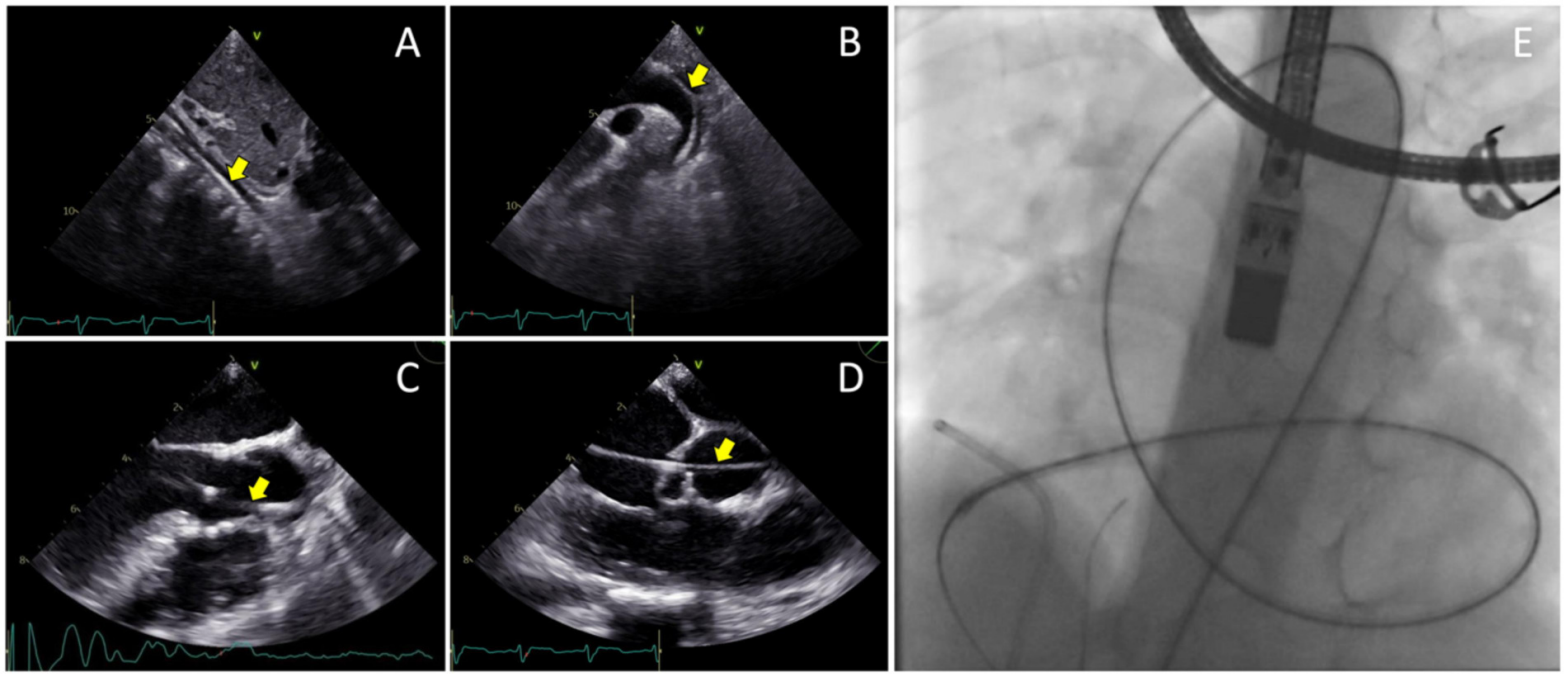
Transesophageal echocardiography views of the 0.035” soft hydrophilic wire (yellow arrows) being advanced from the femoral artery **(A)** into the abdominal aorta, **(B)** through the descending aorta and aortic arch, **(C)** into the left ventricle, **(D)** and crossing the tricuspid valve defect. **(E)** Fluoroscopy view of the Terumo wire after crossing the site of defect.

**Figure 4:**
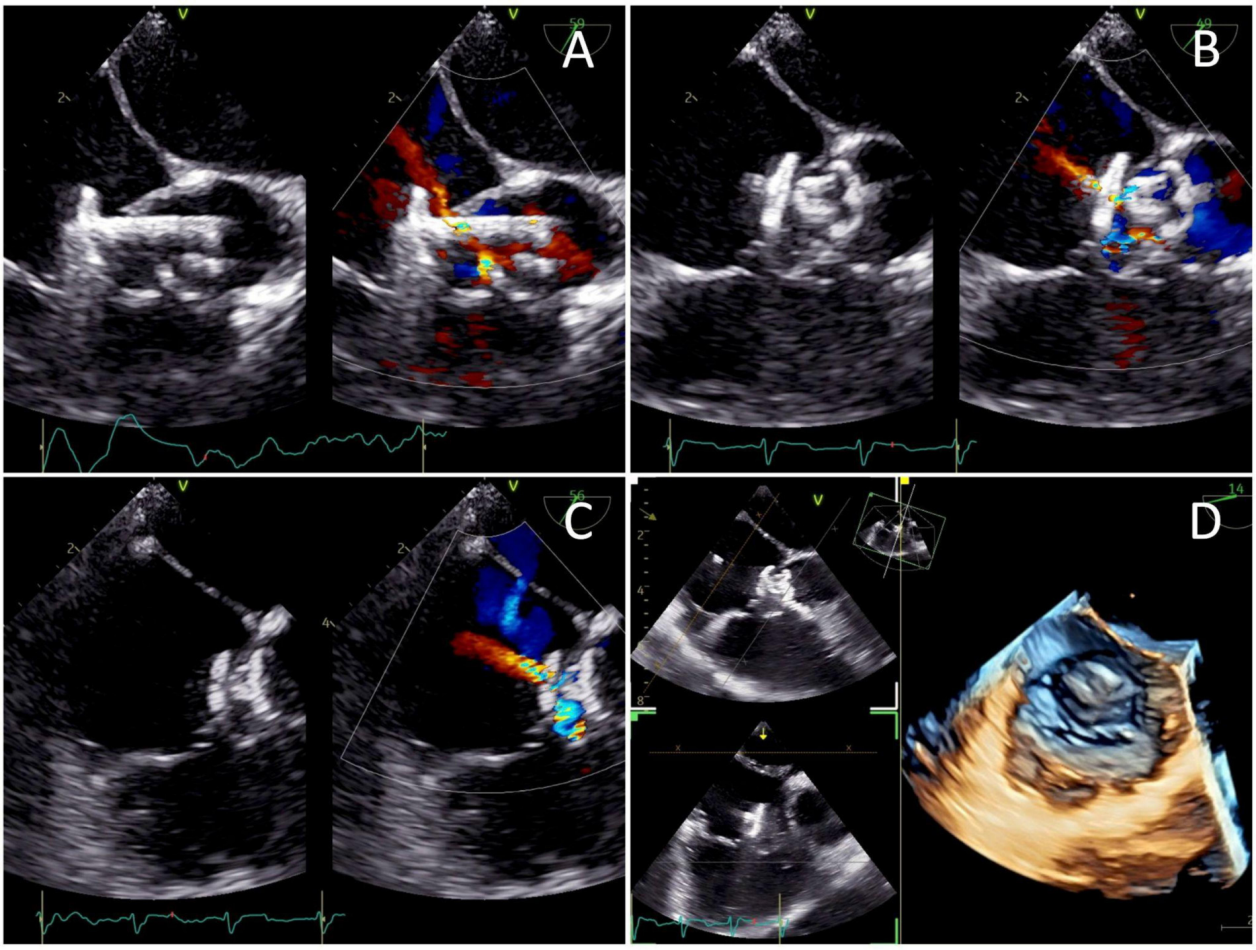
Transesophageal echocardiography views of **(A)** the low-pressured disc being deployed at the right atrium before the whole system was pulled into the tricuspid valve defect, **(B)** the high-pressured disc deployed at the left ventricle ends to seal the defect, and **(C)** a minimal residual central shunt with no peripheral leakage. **(D)** Post-procedure images demonstrating the device successfully sealing the defect.

The femoral vein was punctured, and a 5F sheath was inserted. Guided by TTE and TEE, a JR Guiding catheter 3.5/5F assisted by 0.035” soft hydrophilic wire was introduced from the femoral vein into the inferior vena cava, and into the right atrium. The catheter was directed at the jet turbulence to cross the tricuspid valve defect. Despite several trials, including switching to a Multipurpose side hole catheter, this approach remained unsuccessful. An alternative via the right femoral artery was then attempted. The catheter was inserted through the aorta, to the left ventricle, which successfully crossed the tricuspid valve defect directed at the right atrium, before being advanced further into the inferior vena cava. Konar-MF VSD Occluder No. 6/8 mm was deployed through the catheter and advanced until it reached the tip of the catheter (Figure 3). The low-pressured disc was placed at the right atrium. The whole system was then pulled into the site of the LV-RA connection, and the high-pressured disc was placed at the left ventricle ends to properly seal the defect (Figure 4).

### 2.5 Post-procedure and follow-up

A well-positioned device with minimal residual central shunt and no peripheral leakage was confirmed from the post-procedural TEE (Figure 4). TEE also showed an improved ejection fraction of 70%. The total duration of the procedure was 80 min with an estimated blood loss of 10 mL. The fluoroscopy time was 54 s, and the dose area product (DAP) was 64.41 μGy·m^2^. The patient's hemodynamics were stable throughout and after the procedure, and he was discharged the following day. At 3 months follow-up, the patient presented without symptoms. TTE evaluation revealed a mild trivial TR, with a maximum velocity (Vmax) of 217 cm/s and a pressure gradient of 19 mmHg. The device was still properly stowed in place, without any residual shunt (Supplementary Video 4). There was no evidence of tricuspid stenosis, thrombus formation, endocarditis, or valve degeneration, indicating an overall good postoperative result.

## 3 Discussion

To our knowledge, this is the first pediatric retrograde Konar-MF closure of an LV-RA connection due to a tricuspid valve defect accompanied with a residual MSA. Tricuspid defects, particularly perforations, have largely been associated with pacemaker or defibrillator lead implantation. However, symptoms manifestation from such complications may take up to years after the procedure, making it difficult to diagnose (4). In the present case, the patient was admitted following a surgical VSD closure one year prior. There was no history of intracardiac device implantation, infective endocarditis, or other congenital abnormalities. This leads to the speculation that the tricuspid leaflet defect may be due to an iatrogenic injury from his past VSD correction.

There are alternative etiologies to consider for an LV-RA connection given the absence of echocardiographic data from the previous VSD closure. If the underlying cause is a congenital malformation, then a Gerbode defect is a plausible differential diagnosis due to its overlapping features. According to the classification described by Winter et al. (1), an indirect (type II) Gerbode defect would best fit the patient's condition. This is characterized by an interventricular septal defect located inferior to the tricuspid leaflet, with a tricuspid valve abnormality such as leaflet perforation. However, the pre-procedural TEE in this case demonstrated an isolated tricuspid leaflet defect with a residual MSA, resulting in a direct shunt from the left ventricle to the right atrium without residual VSD. These findings suggest that an iatrogenic tricuspid leaflet defect secondary to the prior surgical intervention is still the most likely explanation.

Surgery remains the established treatment for valve leaflet defects, either through valve replacement or pericardial patch repair (10–12). Similar to other surgical interventions, this carries the risk of complications, particularly to more vulnerable populations such as pediatrics. Advancements in current technologies have allowed percutaneous closure as a more minimally invasive alternative. Unfortunately, reported cases were limited only to the mitral valve and on adult patients (5–9). Compared to a surgical repair, transcatheter closure of heart defects offers notable benefits. Although a higher success rate was observed in open-heart patients, percutaneous closure of heart defects resulted in less intensive care unit and hospital length of stays. Non-surgically-treated patients also require less blood transfusions and significantly shorter ventilation time (13–16).

In the present case, the patient's family refused another invasive surgery, therefore the cardiac team settled on a non-surgical approach via transcatheter closure. This is the first documented use of Konar-MF closure with retrograde transarterial approach on an LV-RA connection. Occluder implantation in heart valves inherently carries risks, however published data on transcatheter tricuspid valve implantation (TTVI) in children is scarce. A review by Sazzad et al. (17), with only a single pediatric case of a 12-year-old patient, demonstrated that device placement may interfere with leaflet motion or result in paravalvular leakage, causing persistent or new tricuspid regurgitation. In another recent study, transcatheter tricuspid valve replacement (TTVR) proved to significantly reduce tricuspid regurgitation, with intraprocedural success rate up to 97.2% (18). Follow-up outcomes showed that patients who underwent TTVR had a 4% mortality rate at 30-days and a 1-year mortality rate of 9%, which is significantly lower compared to a surgical repair (19). Embolization or device migration is also a rare yet serious complication, with 3.6% incidence in post-TTVI reports (17). This is important for devices implanted in soft tissues such as the tricuspid valve leaflet, which makes secure anchoring more difficult. Despite these considerations, post-procedure evaluation of the current case demonstrated favorable outcomes, which aligns with prior reports on isolated transcatheter occluder closure of mitral valve defects (6, 20).

TEE played a pivotal role in providing optimal visualization of the LV-RA connection as well as effectively guiding the wire and catheter to cross the defect. Although transcatheter closure of valve defects is generally performed via an antegrade approach, the retrograde approach helps avoid resistance of the turbulent jet flow. In our case, this facilitated catheter navigation across a small defect located within the highly mobile tricuspid valve leaflet. Moreover, the presence of the patient's MSA enabled the catheter to cross the defect from the left ventricle directly into the right atrium. The Konar-MF VSD Occluder No. 6/8 mm was selected primarily due to its double-disc feature, as well as the limited availability of other devices, such as ASD occluders with particular sizes, at our institution. In addition, its relatively soft structure minimizes potential interference with valvular function.

Extended patient follow-up is crucial to monitor the long-term safety and efficacy of transcatheter device closure of tricuspid leaflet defects. Reports on children with implanted heart valve prostheses have shown an infective endocarditis risk of up to 1.6%. This is highest within the first 6 months after device placement, but late cases after several years may occur (21). As there is currently no large-scale, long-term studies on closure of leaflet defects, regular evaluation is needed to assess the possibility of residual shunts, conduction disturbances, and device embolization.

## 4 Limitations

This report describes a single case with a follow-up period of less than one year. Further studies with larger cohorts and extended follow-up durations are warranted to validate the safety, efficacy, and long-term outcomes of transcatheter tricuspid leaflet repair in pediatric patients.

## 5 Conclusion

Percutaneous transcatheter device closure of an LV-RA connection in pediatrics is a feasible alternative to surgery using a retrograde technique. For long-term outcome, an extended follow-up is necessary to evaluate both the patient's clinical condition and device placement.

## Data Availability

The original contributions presented in the study are included in the article/Supplementary Material, further inquiries can be directed to the corresponding author.
