# Supplementary figures and images for: Case Report: Successful transcatheter repair of left ventricle-right atrium connection following ventricular septal defect surgery
Source: Front Cardiovasc Med. 2026 Jan 16;12:1667869. doi: 10.3389/fcvm.2025.1667869 (PMC12856938; doi:10.3389/fcvm.2025.1667869)

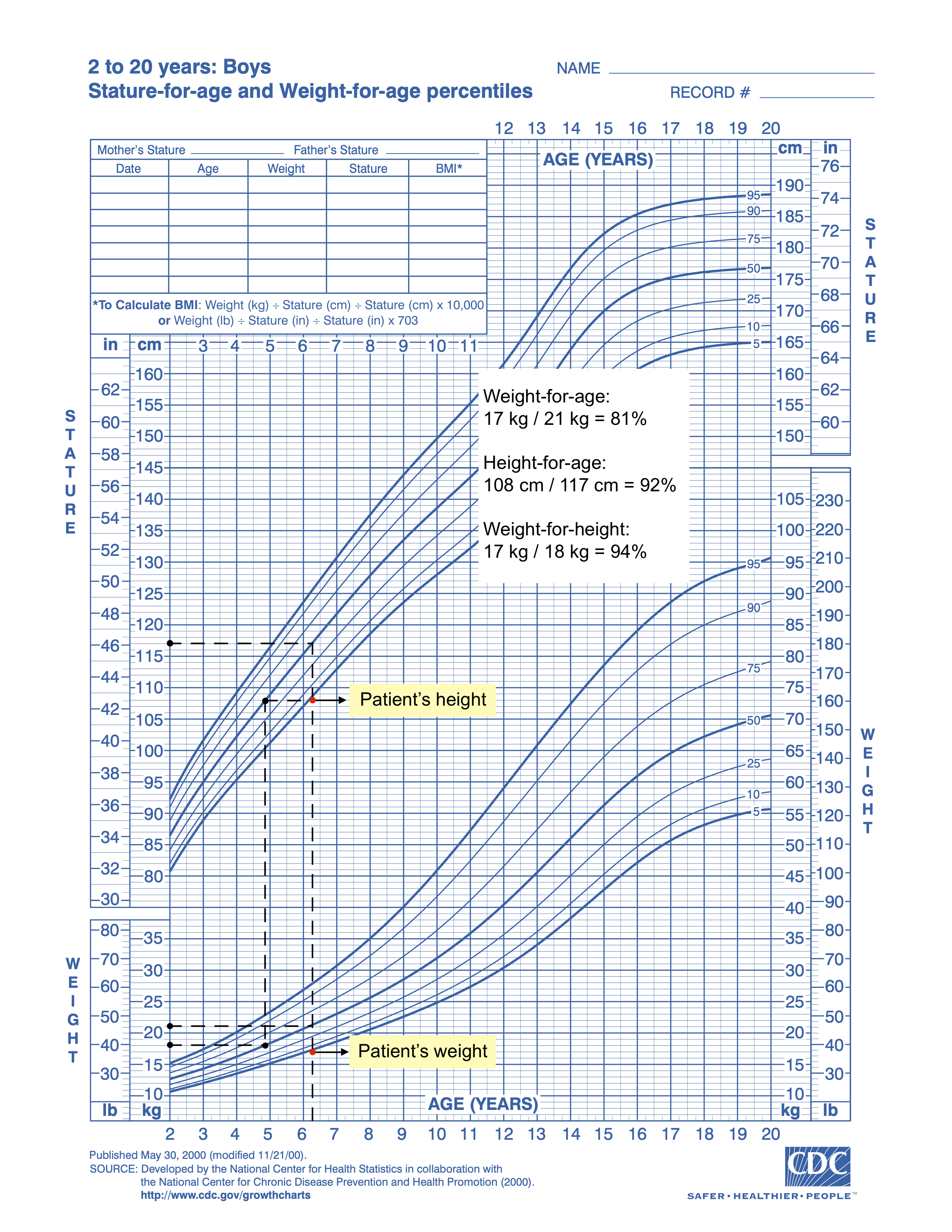

Supplement: Supplementary file 1 [file Image1.tiff]

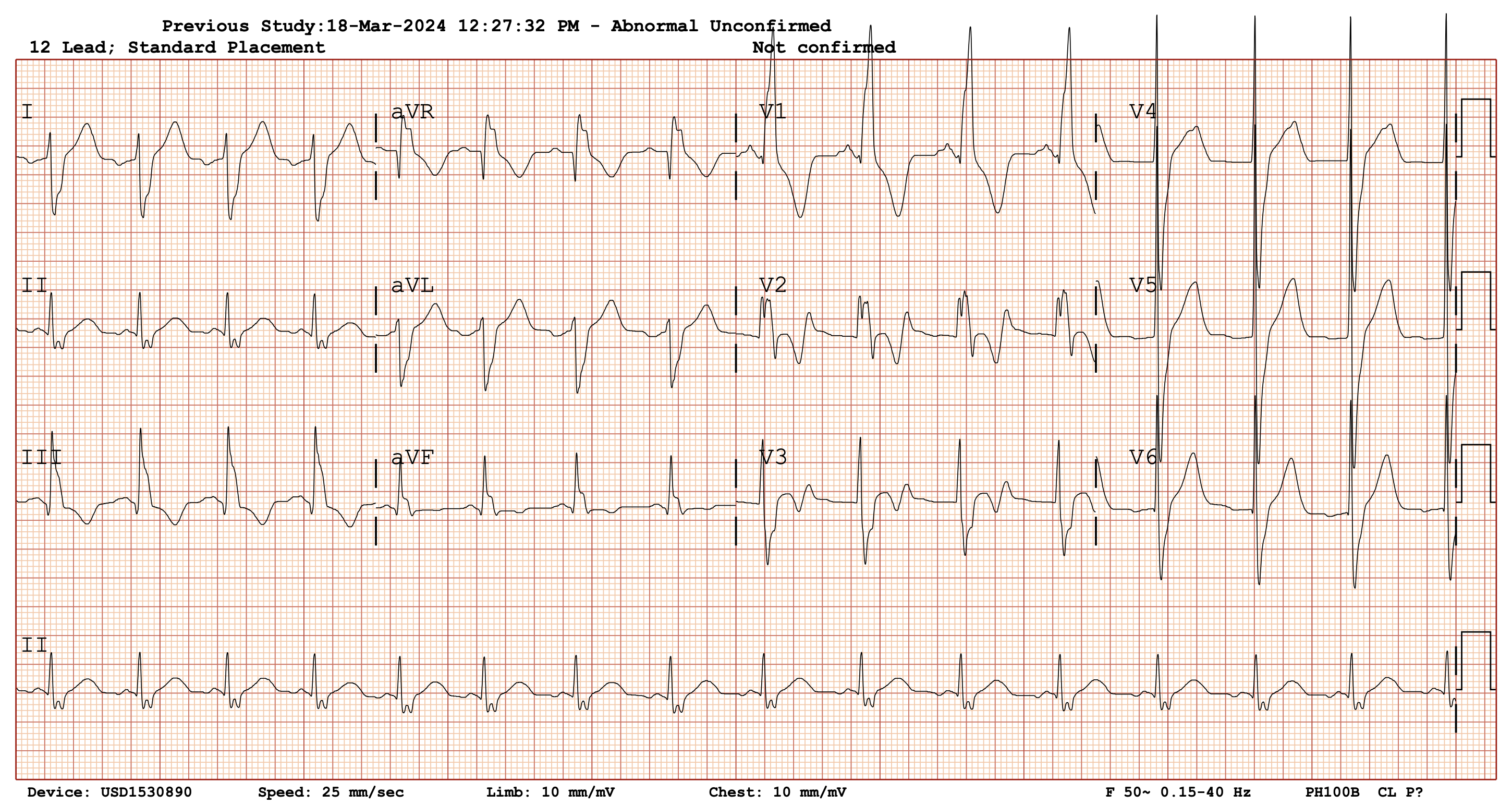

Supplement: Supplementary file 2 [file Image2.tiff]

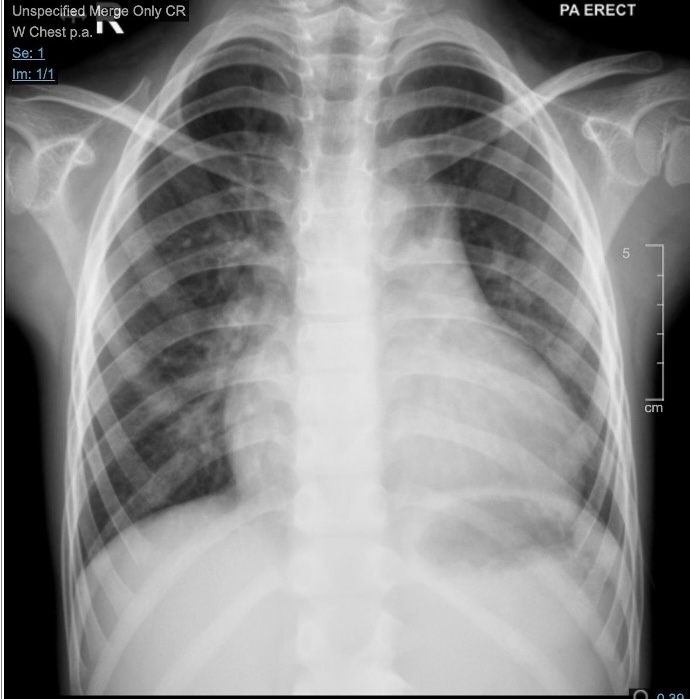

Supplement: Supplementary file 3 [file Image3.jpeg]
